# Promoting osteointegration effect of Cu-alloyed titanium in ovariectomized rats

**DOI:** 10.1093/rb/rbac011

**Published:** 2022-02-24

**Authors:** Xiyue Zhang, Hui Liu, Ling Li, Cuishan Huang, Xiangbo Meng, Junzuo Liu, Xueling Bai, Ling Ren, Xinluan Wang, Ke Yang, Ling Qin

**Affiliations:** 1 Institute of Metal Research, Chinese Academy of Science, Shenyang 110016, PR China; 2 Translational Medicine Research Center, Shenzhen Institute of Advanced Technology, Chinese Academy of Sciences, Shenzhen, PR China; 3 Musculoskeletal Research Laboratory of Department of Orthopaedis & Traumatology, The Chinese University of Hong Kong, HK SAR, PR China

**Keywords:** copper-alloyed titanium alloy, osteoporosis, implant, osseointegration, vascularization

## Abstract

Osteoporosis is a common skeletal disease making patients be prone to the osteoporotic fracture. However, the clinical implants made of titanium and its alloys with a poor osseointegration need a long time for healing and easily to loosening. Thus, a new class of Cu-alloyed titanium (TiCu) alloys with excellent mechanical properties and bio-functionalization has been developed. In this study, the osteoporosis modeled rats were used to study the osteointegration effect and underlying mechanism of TiCu. The results showed that after implantation for 4 weeks, TiCu alloy could promote the reconstruction of vascular network around the implant by up-regulating vascular endothelial growth factor expression. After 8 weeks, it could further promote the proliferation and differentiation of osteoblasts, mineralization and deposition of collagens, and then significantly increasing bone mineral density around the implant. In conclusion, TiCu alloy would enhance the fixation stability, accelerate the osteointegration, and thus reduce the risk of aseptic loosening during the long-term implantation in the osteoporosis environment. This study was the first to report the role and mechanism of a Cu-alloyed metal in promoting osteointegration in osteoporosis environment, which provides a new attractive support for the improvement of future clinical applications of Cu-alloyed antibacterial titanium alloys.

**Figure:**
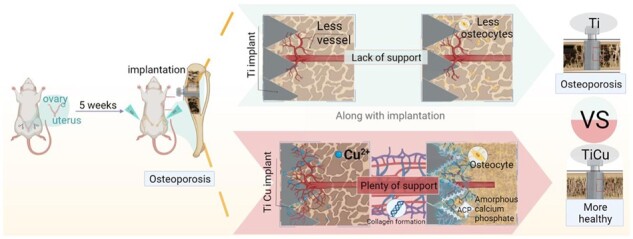

## Introduction

Osteoporosis (OP) is a systemic skeletal disease characterized by low bone mass and degeneration of bone microstructure, which often leads to brittle transformation of bone and high risk of fracture [1, 2]. OP is a common orthopedic disease among the elder people, with an incidence of 15–23% among the citizen over 50 years old in China, which means more than 80 million people in China suffering OP per year [3–5]. More seriously, due to OP, there are about 9 million cases of osteoporosis fracture (OPF) in the world every year, and the main fracture sites are at spine, distal forearm and proximal femur [6].

At present, the effective method in clinical treatment of bone fracture is the internal fixation in order to form a direct and orderly structural and functional connection between implant and bone tissue through the osteointegration effect by implantation of the fixation system, so as to accelerate the bone remodeling process and bone healing of the fracture site [7]. It worth noting that an adequate vascular system at the implantation site can deliver immune cells to the interface, and provide necessary oxygen and nutrition for osteogenic differentiation. Meanwhile, as a mineral deposition template, the vascular system can also regulate bone morphogenesis and play an important role in the improvement of osseointegration environment [8–10]. However, for patients with OPFs, the lack of physiological stimulation via muscle contraction at the fracture site leads to decreases of the vascularity and the function of osteoblasts (OBs). Thus, the process of osteointegration attributed to either osteoconduction or osteoinduction or osteogenesis is lower than that of the normal bone tissue, which finally leads to reduction of bone mass and increase in level of OP. In the fixation of OPFs using metal implants, such vicious cycle may reduce the stability of the bonding at implant–bone interface and induce loosening of the implant that can result in impaired healing in OPF and refracture as well [11–13]. Although the uses of titanium (Ti) and its alloys (such as Ti6Al4V alloy) in orthopedic clinics have demonstrated a great success in the treatment of trauma-induced fractures of normal bones, the lack of vascularization and osteoinduction of these bioinert implants can make them poor osteointegrations in the osteoporotic patients without achieving satisfactory osteogenesis. Therefore, it is more difficult to recover after operation, and the aseptic loosening is easily to occur, which affects the long-term stability of implants as well [14, 15].

Therefore, there are great clinical needs to develop new internal fixation implants for bone fractures, specially to target the challenged OPFs [16]. Medical metals, as the upstream of this special internal fixation implants for bone fracture, should have the features of both structural (load-bearing) and functional (vascularization and osteoinduction) abilities, toward effectively fixing the OPF, shortening the healing time and reducing the potential risk of re-fracture [14, 15, 17].

Copper (Cu) is a unique metal that is not only a commonly used alloying element in metals but also an essential trace metal element in human body that plays important roles in maintaining human health. It is noteworthy that Cu can regulate the expression of related genes in the process of angiogenesis and bone remodeling [18, 19]. Relevant study [20] showed that, compared with bare Ti6Al4V alloy, a Cu-rich diamond-like (Cu/a-C: H) film coated on Ti6Al4V alloy surface could significantly up-regulate the expressions of vascular endothelial cell growth factor (VEGF), alkaline phosphatase (ALP), osteocalcin (OCN) and other growth factors, which expressively promoted the proliferation of vascular cells and enhanced osteogenic activity in rabbits. A CoCrMo-2wt%Cu cobalt alloy could markedly improve the bone-implant contact and mineral apposition rate (MAR) of the implant by promoting the expressions of bone morphogenetic protein 2 (BMP-2) and insulin-like growth factor I, and ultimately effectively accelerate the reconstruction of bone tissue around the implant [21]. A TiCuN film was deposited on 316 stainless steel, and it was confirmed that the film could up-regulate the mRNA expressions of nitric oxide synthase and VEGF, which then promoted the human umbilical vein endothelial cells (HUVECs) proliferation and angiogenic ability [22].

By taking advantage of the above biological functions of Cu, a new 5 wt% Cu-alloyed medical titanium alloy (TiCu) with excellent mechanical properties, corrosion resistance, antibacterial activity and biocompatibility has been developed [23–26]. In the *in vitro* immersion experiment of TiCu, we found that the release amount of Cu ions detected in the solution was about 1.164 μg/cm^2^/day [2]. Liu *et al.* [24] implanted TiCu dental implants in beagles and found that the content of free Cu ions in the main organs and serum of the beagles was in the safe range after 3 months’ implantation. Our research group further analyzed the existence form and release mechanism of Cu in TiCu alloy passivation film and found that an oxygen-deficient TiO_2__−__*x*_ barrier layer with *n*-type semiconducting character could be formed on the surface of TiCu alloy. Cu segregated as Cu interstitials (${\mathrm{Cu}}_{i}^{+}$) in the TiO_2__−__*x*_ barrier layer and was substituted as ${\mathrm{Cu}}_{Ti}^{x'}$. Ultimately, when TiCu was in contact with body, due to the potential difference between these two interfaces, the segregated Cu in the form of Cu ions preferentially passed through the passive film via the Esaki–Tunnelling effect and released into body fluids as a long and steady process [27, 28]. Under the combined action of free Cu ion and TiO_2__−__*X*_ passivation film on the surface of TiCu and$\;{\mathrm{Cu}}_{Ti}^{x'}\;$cation, the preliminary study confirmed that TiCu alloy has excellent vascularization and osteogenic properties and maintain good biosafety. Compared with pure Ti, TiCu alloy could meaningfully up-regulate both osteogenic genes, such as ALP, osteopontin (OPN), OCN and type I collagen (Col I), and vascularization related genes, such as local adhesion spot kinase, matrix metalloproteinase 2 and kinase insertion region receptor [23, 29, 30]. Animal experiments by implanting TiCu alloy into the mandible of healthy Beagles also verified that TiCu could inhibit the bone resorption and form compact cancellous bone tissue around the implant, which accelerated the osteointegration around the implantation site [30]. Therefore, this study was aiming to focus on a severe environment, in the OP modeled animals, to see if this novel TiCu could still have promotion effect on the osteogenesis and angiogenesis and explore the underlying mechanism in other to develop a biologically active and effective fixation implant material for accelerating healing of OPF.

The ovariectomized (OVX) rats were used as it was the most classic and widely used animal model in OP research [31]. Estrogen can maintain the normal bone structure and bone remodeling by inducing endothelial vascular dilation, promoting maturation of OBs for facilitating mineralization, inhibiting formation of osteoclasts and inducing their apoptosis [32, 33]. OVX surgery can rapidly reduce the estrogen level in rats, impair the balance between bone formation and resorption toward the bone resorption in bone remodeling process, which could lead to bone mass reduction and microstructure deterioration [32, 34], and ultimately result in the OP.

In this study, OVX was performed to establish an OP model in 6-month-old female rats, and Cu-alloyed (TiCu) screws were implanted in the bilateral tibias while pure Ti screws were used as the control. The osteointegration ability of TiCu implants was evaluated from the aspects of biomechanics, vascular regeneration and bone remodeling by means of pull-out force test, hole volume analysis after screw extraction, Micro-CT imaging, fluorescence staining, histological analysis and immunohistochemical staining. Then, the location distribution and interaction among the blood vessel network, collagen fibers and new bone tissue around the implanted screws at different time points were investigated. By comparing with normal osteointegration process, the role of TiCu alloy in bone remodeling in OVX rats was elucidated and the related mechanism was explored in order to provide scientific evidences for a new strategy of developing internal fixation implants for the OPF treatment.

## Materials and methods

### Materials

A TiCu alloy with 5 wt% addition of Cu was prepared by melting high purity (>99.9%) Ti and Cu in a 30 kg vacuum consumable arc furnace, and then the ingot was forged and hot-rolled into bars, followed by a heat treatment at 740°C/1 h/air cooling. Preliminary studies showed that the microstructure of TiCu alloy after heat treatment was isometric α-Ti phase with precipitation of granular Ti_2_Cu phase, with good combination of tensile strength (597 ± 3.1 MPa), elongation (26% ± 3.5%), yield strength (457 ± 7.0 MPa), Vickers hardness (215 ± 8.5HV), corrosion resistance and antibacterial performance [23–27, 30, 35].

**Figure 1.**
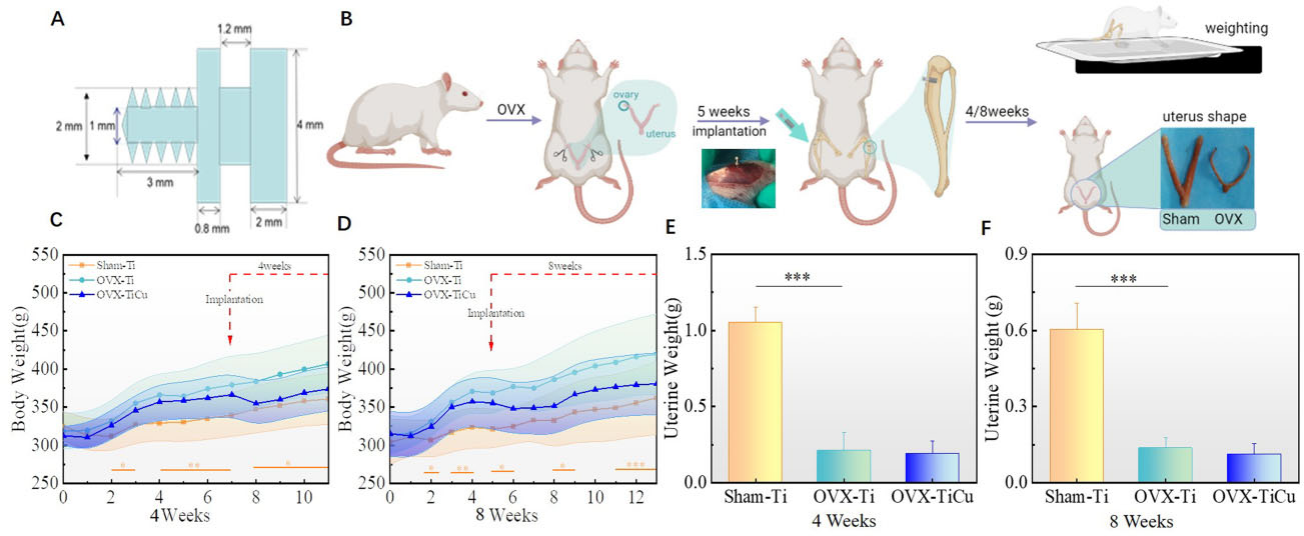
General background information: (**A**) dimension of implant screw; (**B**) surgery procedure and image of the uterus; (**C** and **D**) body weight; and (**E** and **F**) uterine weight after surgical implantation for 4 and 8 weeks. Data were expressed as mean±SD. *n* = 7 for Week 4 group, and *n* = 8 for Week 8 group in panels (D) and (F). ^***^P <* *0.001

Commercial pure titanium was used as the control material. Bone screws for the study were machined by the above pure Ti and TiCu alloy. The dimension of screws was shown in Fig. 1A, 7 mm of total length, 4 mm of upper width and 2 mm of width and 3 mm of length for the thread part.

### Establishment of OVX model

A total of 49 Sprague Dawley female rats, with age of 6 months and an initial weight of 240 ± 10 g, were used as experimental animals from Beijing Charles River Company, China. These rats were raised under the SPF grade condition, and all the operations were carried out in strict accordance with the requirements of Animal Experiment Ethics Committee of Shenzhen Institute of Advanced Technology, Chinese Academy of Sciences. The IACUC number was ‘SIAT-IACUC-190730-YGS-LL-A0867’. The experimental process was illustrated in Fig. 1B.

OVX was performed to establish osteoporotic rat model. After adaptive feeding for 1 week, rats were randomly divided into two groups: OVX group of 34 rats and Sham group of 15 rats. The OVX operation was carried out with 2% pentobarbital sodium (Merck, Germany) for intraperitoneal anesthesia at concentration of 40 mg/kg. After anesthesia, a longitudinal incision of about 1.5 cm was cut along the midline between the 12 ribs and the iliac part of the rat, the subcutaneous tissue was separated passively to the outside, then the muscle layer and peritoneum were separated, and finally the fallopian tubes and blood vessels were ligated at the lower end of the ovaries with Grade 4-0 suture (Shanghai Pudong Jinhuan Medical Supplies Co., Ltd, China). The ovary was removed, and the muscularis and peritoneum were sutured. The contralateral ovaries were removed in the same way. The same skin incision was made on the back of Sham group rats using the same surgical approach, in which only the adipose tissue was removed around the ovary in the same volume as the ovary, and then the wound was sutured.

### Screw implantation and sample collection

#### Implantation

At 5 weeks after establishment of the OP model, pure Ti or TiCu alloy screws were implanted in the proximal bilateral tibiae of animals in OVX group, and pure Ti screws were implanted at the same position in Sham group. Subsequently, the effect of TiCu screw on the bone reconstruction around the implant was studied at fourth and eighth weeks, respectively, after the implantation. The experimental groups were named OVX-Ti (4/8 weeks, *n* = 7/8, *n* is the number of experimental rats), OVX-TiCu (4/8 weeks, *n* = 7/12) and Sham-Ti (4/8 weeks, *n* = 7/8). The screw implantation was conducted as follows: the rats were anesthetized by intraperitoneal injection of 2% pentobarbital sodium (40 mg/kg) and placed in supine position. A 1.6 or 1.8 mm surgical drill (MNT998512, Shanghai Minnet Industrial Co., Ltd, China) was used to vertically drill into 2 mm depth at 2 mm below the medial tibial growth plate on hind legs of rat, and then a screwdriver was utilized to twist the screw into the hole. The wound layer was closed layer by layer after marking. Injection of penicillin sodium (Guangzhou Baiyunshan Tianxin Pharmaceutical Co., Ltd, China) (100 000 units/kg) was conducted for three consecutive days to prevent infection on the surgical site. The weight of rats was recorded every week.

#### Sampling

Microfil perfusion was performed into the vascular system of rats at 4 weeks after implantation to obtain an accurate vascular skeleton image. The rats were anesthetized and injected with 2 ml of 600 U/ml heparin sodium (Beijing Coolaber Technology Co., LTD, China) normal saline. At 2 min later, the abdominal cavity was opened, and vascular cannula tubes were inserted into the distal ends of the arteries and veins, respectively. Then, a peristaltic pump (Longerpump, UK) was used to continuously irrigate 60 U/ml heparin sodium normal saline at 10 rpm (arteries in and veins out). After the blood was completely rushed out, the contrast agent (19 ml MV-diluent and 25 ml MV-117 Orange) and the catalyst (2.5 ml MV Agent) were injected at a constant speed. The tube was ligated, the abdominal cavity was sutured, and the rats were stored overnight at 4°C. At 12 days before sampling of the rats after 8 weeks of implantation, xylenol orange (80 mg/kg) was subcutaneously injected, and after 10 days, calcine green (25 mg/kg) was subcutaneously injected to study new bone formation and remodeling by evaluating its apposition rate.

At fourth and eighth weeks of implantation, the bilateral tibiae of each group were harvested, respectively. Then the external soft tissues were collected and cleaned before they were fixed in 10% neutral formalin (Sangon Biotechnology (Shanghai) Co., Ltd, China). The left tibia was used for biomechanical testing, and the right tibia was used for Micro-CT scan and subsequent histological analysis. The uterus was collected, weighed and recorded.

### Micro-CT analysis

**Figure 2.**
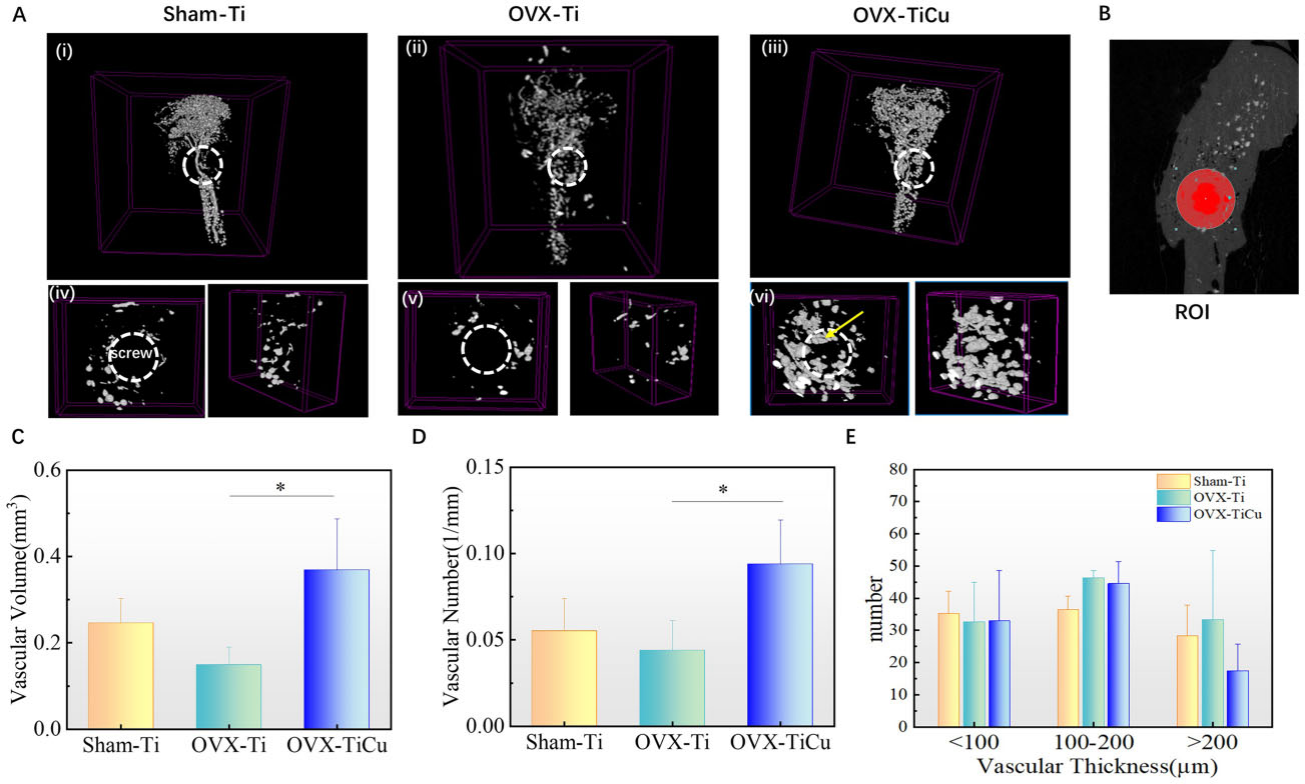
Micro-CT analyses of bone vessels after 4 weeks of screw implantation: (**A**) micro-CT scan modeling; (**B**) ROI for analysis; and (**C**–**E**) quantitative analysis results of volume, number and thickness distribution of blood vessels in ROI. The data were expressed as mean±SD. *n* = 7 in panels (C) and (E). ^*^P <* *0.05

Samples harvested after implantation for 4 and 8 weeks, and those from the right tibia of rats were decalcified in a 37°C EDTA decalcification solution (Boster Biological Technology Co., Ltd, USA). Micro-CT scanning was performed to analyze the vascular tissue of rat tibia and the bone tissue around the screw. CTan software was used for reconstruction analysis on the tibias of rats after 4 weeks implantation. Around the surface where the screw contacted to the bone, a cylinder with diameter of 2 mm and depth of 1.5 mm was selected as the region of interest (ROI), as shown in Fig. 2B, with total 167 layers. Different thresholds were determined according to each sample, and in ROI, the volume and number of blood vessels were statistically analyzed. Besides, in order to evaluate whether TiCu implants affect the distribution of vascular thickness, 100 blood vessels were randomly selected in ROI and the number of blood vessel thickness distributed within 100, 100–200 and >200 μm were statistically analyzed [36–38].

**Figure 3.**
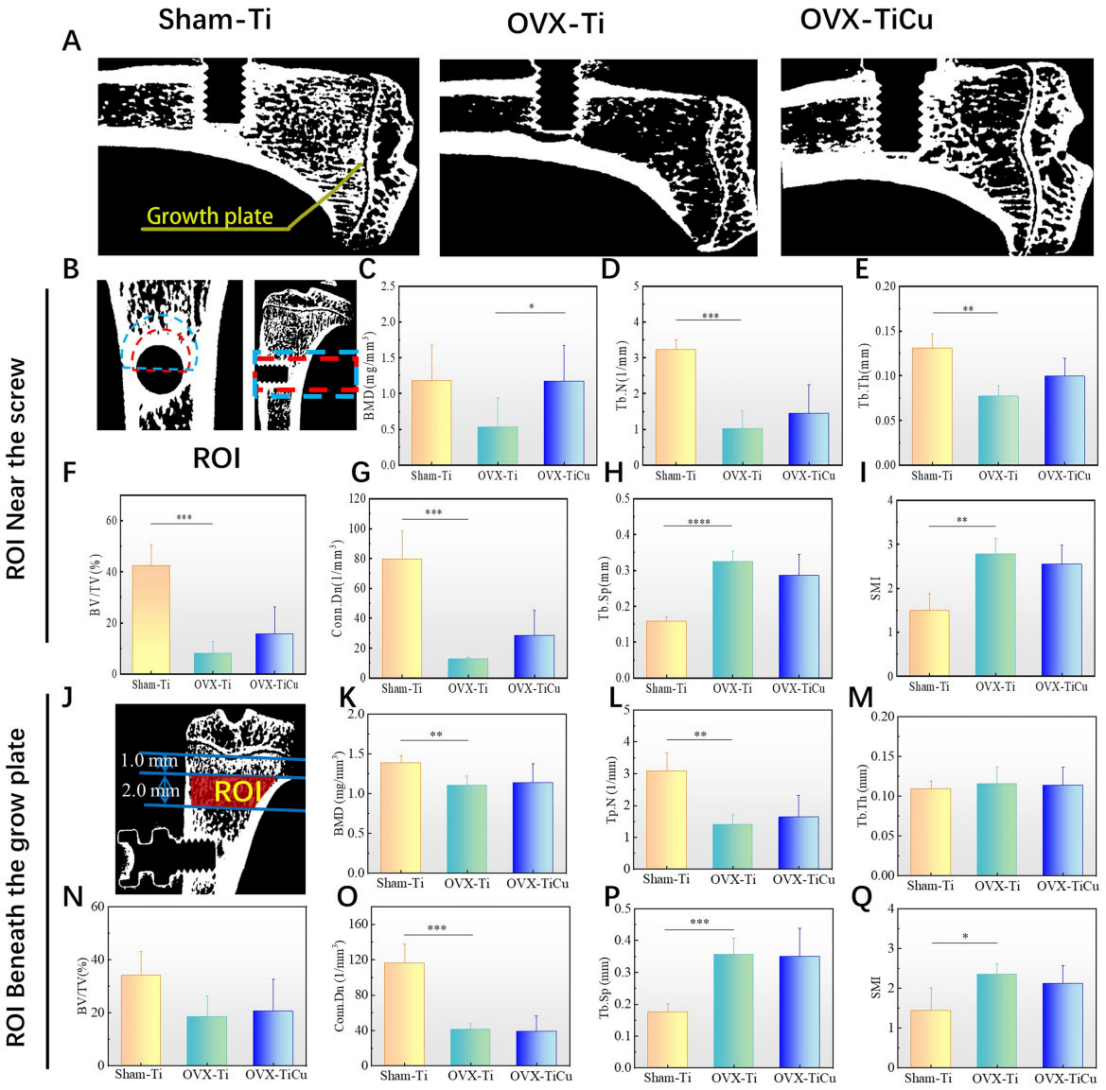
Micro-CT analyses of bone structure after 8 weeks of screw implantation: (**A**) micro-CT scans on Sham-Ti, OVX-Ti and OVX-TiCu groups, respectively; (**B** and **J**) ROI for analyses; and (**C–Q**) quantitative analyses of bone structure parameters. Data were expressed as mean±SD. *n* = 8 in panels (C) and (I). ^*^P <* *0.05, ^**^P <* *0.01, ^***^P <* *0.001

Ctvox and Dataviewer software were used to quantitatively analyze the bone mass parameters and bone structure parameters of rat tibia 8 weeks after implantation. Taking the side of the screw close to the bone marrow cavity as a semicircle with radius of 2 mm, the artifact (red semicircle) was removed, and then a semicircle with radius of 3 mm (blue semicircle) was made. The ring between red and blue dotted lines was the ROI for analysis (Fig. 3B). Bone mass parameters and bone structure parameters included bone volume fraction (BV/TV), bone mineral density (BMD), trabecular thickness (Tb. Th), trabecular spacing, trabeculae number (Tb. N), mean connection density (Conn. Dn) and structural model index (SMI).

### Biomechanical analysis

#### Pull-out force

**Figure 4.**
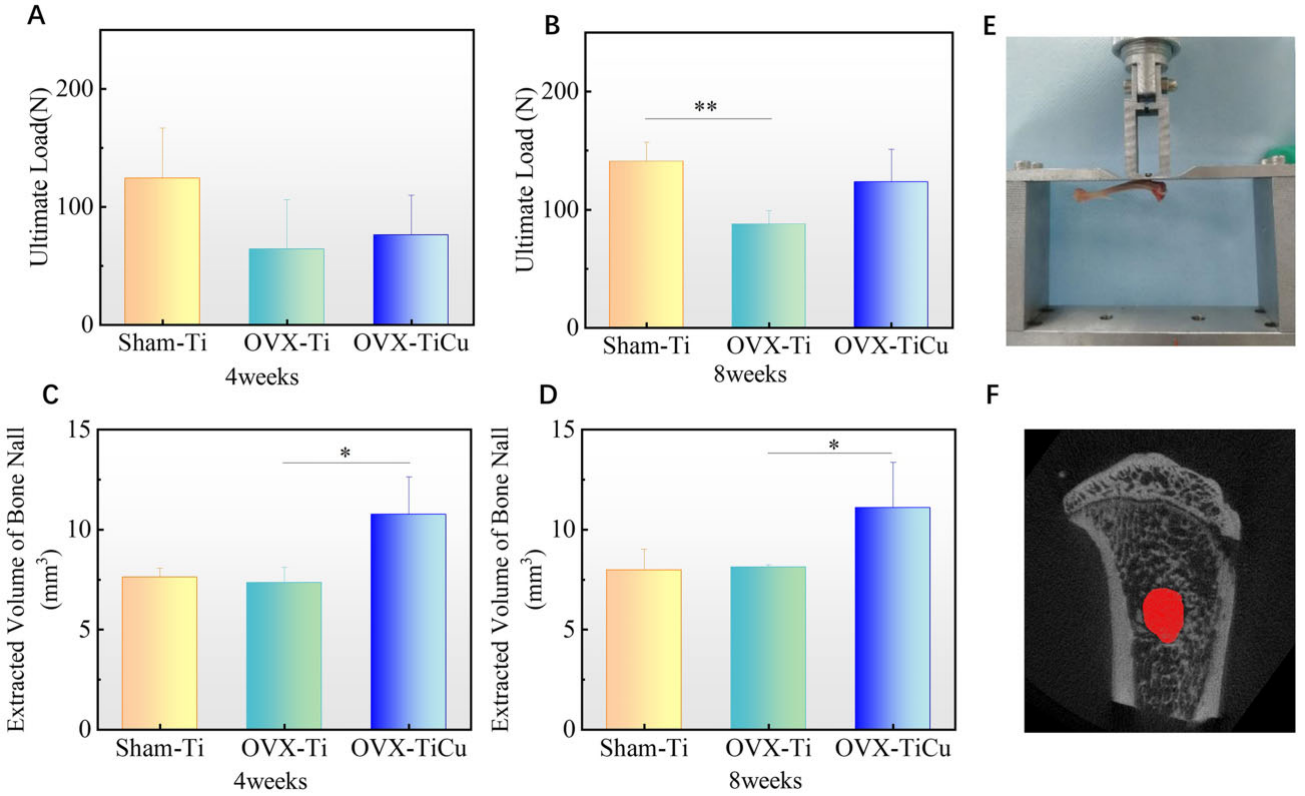
Results of biomechanical experiment: (**A** and **B**) ultimate load of pull-out of samples after 4 and 8 weeks of screw implantations, respectively; (**C** and **D**) volumes of holes left after pulling out of screws with 4 and 8 weeks of implantations, respectively; (**E**) cross-head of biomechanical testing machine; and (**F**) ROI for analysis. Data were expressed as mean±SD. For 4 weeks *n* = 7, and for 8 weeks *n* = 8 in panels (A) to (D). ^*^P <* *0.05

As shown in Fig. 4E, a biomechanical testing machine (MX-0350, Machine Industrial System Co., Ltd, China) was used to measure the pull-out force at the contact interface between the screw with attached new bone tissue and the residual bone of the left tibia. The soft tissue on the external contact surface between the screw and the bone was removed, and the screw was fixed with a clamp. During the experiment, the screw was pulled upward at a constant speed of 1.00 mm/min. During this process, the ultimate force in the process of screw loosening was detected as the maximum binding force at interface of screw-new bone.

#### Hole volume

In order to quantitatively analyze the amount of bone taken away after the screw removal and to evaluate the adhesion between screw and surrounding bone tissue, Micro-CT scanner (Skyscan1176, Bruker, Belgium) was employed to examine the samples after pull-out test. In the Ctan software, the hole surface was selected as the ROI (Fig. 4F), the thresholding was set as 0–48, and the 3D analysis was conducted. The BV value obtained was the hole volume.

### Histological analysis

#### Hematoxylin-eosin staining

In order to observe the tibial histological appearance, after the Micro-CT scan, the screws of 4 weeks implantation were soaked in EDTA for decalcification. After the redundant organization was eliminated, samples were cleaned by running water for 24 h, dehydrated by graded ethanol (Sinopharm Chemical Reagent Co., Ltd, China), hyalinized by xylene (Sinopharm Chemical Reagent Co., Ltd, China) and finally embedded by paraffin (Leica, Germany). The section thickness was 5 μm, and the section direction was parallel to the long axis of tibia.

Hematoxylin-eosin (H&E) staining was used to observe the morphology of bone trabecular tissue. H&E staining sections were stained with Regaud hematoxylin (Proteintech Group, USA) and returned to blue with 0.2% ammonia solution. After dyed with Eosin (Beyotime Biotechnology Co., LTD, China), samples were dehydrated by gradient ethanol, fixed with xylene and finally sealed with neutral resin (Shanghai Aladdin Biochemical Technology Co., Ltd, China). The ROI was selected as a rectangle with a wide edge extending 400 μm from the teeth tip to the bone trabecula, and a long edge with 1330–1340 μm length. The sections were observed and imaged by using Leica DMI8 (Leica, Germany) inverted fluorescence microscope. Trabecular bone area (Tb. Ar) was quantitatively analyzed by using Image Pro Plus.

#### Masson staining

Masson staining was employed to observe the collagen production and distribution at the contact surface between the screw and bone tissue. After sectioning as same as Hematoxylin-eosin staining section, the sections were dyed with hematoxylin dye, redyed with Masson Deli Chunhong acid reddish Staining Solution (Solarbio, China), immersed and differentiated in 2% glacial acetic acid (Servicebio, China) and 1% phosphomolybdic acid (Servicebio, China), then stained with aniline blue (Servicebio, China), leached by 2% glacial acetic acid, dehydrated by ethanol and hyalinized by xylene, finally sealed with neutral gum and observed under an inverted fluorescence microscope.

#### Fluorescence labeling

Xylenol orange-calcein double fluorescence signing was detected to label the new bone formation at the screw-bone interface after 8 weeks implantation. In brief, tibial samples were dehydrated with gradient ethanol, washed with xylene and embedded with methyl methacrylate. Sectioning was performed using a hard tissue slicer (EXAKT E300CP, Exakt Vertriebs Gmbh, Germany) along the sagittal plane of the vertical screw with a thickness of 100–300 μm. The sections were observed using Leica DMI8 red and green filters inverted fluorescence microscope. Three internal teeth triangles near the growth plate side and the site of 250 μm extension were selected as the ROI. The bone formation parameters were quantitatively analyzed by Image Pro Plus, including MAR, fluorescence labeled perimeter percentage (%L. PAM) and new bone formation rate on bone surface (BFR/BS).

#### Immunohistochemical staining

In order to evaluate the expression of endothelial cell growth factor (VEGF) on screws implanted in bone for 4 weeks, the embedded soft tissue sections were immersed in a frozen antigen repair solution (Beyotime Biotechnology Co., LTD, China), and incubated with hydrogen peroxide without light to remove the endogenous catalase. The non-specific sites were sealed with 3%BSA. After that, anti-VEGF (Abcam, UK) and anti-rat secondary antibody (Beyotime Biotechnology Co., LTD, China) were added, followed by incubation at room temperature. Then DAB was used for color rendering, hematoxylin for re-dying, and 0.2% ammonia water for returning to blue. Finally, samples were dehydrated by gradient ethanol, and sealed with neutral resin. All the inter-tooth triangles on both sides of teeth tip with an extension of 600 μm length toward the trabecular bone were selected as the ROI. Images were observed and collected under a microscope, and the proportion of VEGF positive area was quantitatively analyzed using Image Pro Plus.

### Statistical analysis

The results were expressed as mean ± standard deviation (SD). Statistical significance was analyzed by unpaired *T* test of prime software, which was used to compare the *P*-values of OVX-Ti and OVX-TiCu, Sham-Ti and OVX-Ti groups, and the differences were considered statistically significant when ^*^P <* *0.05.

## Results

### General observation

After OVX surgery, and before and after bone-screw implantation, the consumption of rat diet was normal in each group. The operation sites healed well, without obvious swelling and exudation as well as abnormal signs, such as chills and high fever in the rats. After OVX, the weight of rats in each group showed an upward trend (Fig. 1C and D), and the weights of rats in OVX-Ti and OVX-TiCu groups were always higher than that in Sham-Ti group. Before and after screw implantation, the body weight of rats in each group did not show significant changes, indicating that the normal physical function of the rats was not affected before and after screw implantation.

Figure 1E and F showed that the uterine weights of OVX-Ti group and OVX-TiCu group were significantly lower than that of Sham group (*P *<* *0.05), indicating that the ovaries removal resulted in a uterine atrophy (Fig. 1B), confirming successful establishment of osteoporotic model in rats.

### Imageology analysis

#### Vascular microimaging

Micro-CT scanning was performed on the samples after Microfil angiography. The 3D microscopic images (Fig. 2A) showed that capillaries were formed around the screws in each group after 4 weeks implantation (white circle). The top view (Fig. 2A(iv–vi)) showed that the peripheral vascular tissue of OVX-TiCu group extended into the screw implantation site and interacted with the screw (yellow arrow). Through the quantitative analysis on the microstructural characteristics of blood vessels in the ROI, which was 1 mm wide and 1.5 mm deep around the screw, it was calculated that the number and volume of blood vessels around the screw in OVX-TiCu group were 0.09 ± 0.02/mm and 0.36 ± 0.11 mm^3^, respectively, which was twice more than those around the screw in OVX-Ti group (0.04 ± 0.02/mm and 0.15 ± 0.04 mm^3^) (Fig. 2C and D). This indicates that in the osteoporotic animal model, TiCu screw could better promote the angiogenesis than pure Ti screw, and then accelerate the process of vascular reconstruction around the screw, which attributed to the biological function of Cu ions released from TiCu alloy according to the previous study. According to the statistics of the number distribution in different average vascular wall thickness within the ROI, as shown in Fig. 2E, there was no significant difference in per thickness range among the groups.

#### Microscopic imaging of bone tissue

After screw implantation over 8 weeks, the bone tissues around the screw and beneath the growth plate were reconstructed by Micro-CT scanning, respectively. The bone mass and bone structure parameters in the ROI (on the growth plate side, 1.5–3.0 mm away from the screw/1.0–3.0 mm beneath the growth plate) were analyzed quantitatively. The scanning results (Fig. 3A) showed that, compared with OVX-Ti group, OVX-TiCu group had more bone mass, compact cancellous bone structure and less perforated ‘mesh’ in trabecular plate structure, meaning that there should be a better osteointegration around the TiCu screw. Compared with Sham-Ti group, OVX-Ti group showed osteoporotic bone structure and significantly reduced bone mass around the screw. Quantitative analysis around the screw (Fig. 3C–G) also confirmed that BMD, Tb. N, Tb. Th, BV/TV and Conn. Dn (1.18 ± 0.49 mg/mm^3^, 1.45 ± 0.79/mm, 0.10 ± 0.02 mm, 15.70% ± 10.68% and 28.52 ± 16.85/mm^3^) in OVX-TiCu group were significantly higher than those in OVX-Ti group (0.53 ± 0.41 mg/mm^3^, 1.02 ± 0.49/mm, 0.08 ± 0.01 mm, 8.19% ± 4.63% and 12.66 ± 1.12/mm^3^). The BMD value of OVX-TiCu group was much higher than that of OVX-Ti group (*P *<* *0.05), indicating that TiCu alloy greatly improved the bone mass and bone structure parameters, which was conducive to improve the bone strength around the screws. The parameters of OVX-Ti group were also significantly lower than those of Sham-Ti group (1.18 ± 0.50 mg/mm^3^, 3.22 ± 0.27/mm, 0.13 ± 0.02 mm, 42.40% ±8.31% and 79.72 ± 18.62/mm^3^), indicating that OVX could significantly reduce the bone density and make a prominent OP phenotype of mice. Notably, the BMD value of OVX-TiCu group has recovered to the level of Sham-Ti group, and other indices were between OVX-Ti group and Sham-Ti group, indicating that TiCu alloy should have great beneficial effect for applications in the osteoporotic patients.

The quantitative parameters measured in the range of 1.0–3.0 mm below the growth plate (Fig. 3K–Q) indicating the ectopic bone formation condition after screw implantation. The results showed that BV/TV, Tb. N and BMD (20.55% ± 12.14%, 1.64 ± 0.68 and 1.14 ± 0.24) in OVX-TiCu group were also higher than those in OVX-Ti group (18.62% ± 1.69%, 1.41 ± 0.30 and 1.10 ± 0.11), even though the position was far from the implantation site. It is worth noting that the SMI is a parameter describing the degree of plate and rod-shape of trabecular bones. The SMI of healthy bone structure is close to 0, meaning that the bone trabeculae are mainly lamellar structure. Conversely, SMI of the osteoporotic bone structure is close to 3, meaning that the bone trabeculae are mainly rod structure. A larger SMI value reflects a greater degree of bone trabecular transformation from plate to rod, i.e. a higher degree of OP. As shown in Fig. 3I and Q, SMI indexes of OVX-TiCu group were 2.37 ± 0.40 and 2.12 ± 0.45, those of OVX-Ti group were 2.78 ± 0.35 and 2.35 ± 0.27, and those of Sham-Ti group were 1.49 ± 0.39 and 1.44 ± 0.56. This indicates that compared with Sham-Ti group, bone trabeculae structures in OVX-TiCu and OVX-Ti groups were all rod-shape, while the degree in OVX-TiCu group was lower than that in OVX-Ti group, i.e. TiCu screw was conducive to reducing the degree of OP.

### Biomechanics

#### Pull-out force

The maximum pull-out forces of the screws were measured using a biomechanical testing machine, and the results were shown in Fig. 4A and B. The maximum pull-out forces of OVX-TiCu, OVX-Ti and Sham-Ti groups were 75.98 ± 33.99 N, 63.97 ± 42.20 N and 124.26 ± 42.66 N, respectively, after implantation for 4 weeks, and reached 123.27 ± 28.00 N, 87.65 ± 11.50 N and 140.83 ± 16.30 N after 8 weeks implantation. At both time points, the average binding force of screws in OVX-TiCu group was higher than that in OVX-Ti group, and former increased larger than the later with increase of the implantation time. BMD is one of the main affecting factors for pull-out force of the screws. In the OVX model, the BMD around TiCu screw was significantly higher than that around pure Ti screw. Thus, the bone bonding effect of TiCu screw was obviously enhanced. The maximum pull-out force of screws in the OVX model was much lower than that in Sham-Ti group at 4 weeks implantation, but the difference was reduced after 8 weeks implantation, especially the OVX-TiCu group, indicating that the osteoporotic bone tissue in the OVX model could largely reduce the implantation stability of the screws, but the degree of OVX-TiCu group could be alleviated.

#### Hole volume

The quantitative results of the hole volume left on the tibia after pulling out of screws were shown in Fig. 4C and D. After 4 weeks implantation, the average hole volumes of OVX-TiCu, OVX-Ti and Sham-Ti groups were 10.75 ± 1.90 mm^3^, 7.34 ± 0.77 mm^3^ and 7.61 ± 0.47 mm^3^, respectively. After 8 weeks implantation, these values became 11.09 ± 2.28 mm^3^, 8.12 ± 0.12 mm^3^ and 7.98 ± 1.04 mm^3^, respectively. It could be seen that the hole volume left on tibias of OVX-TiCu group was obviously larger than that of OVX-Ti group. This means that in the OVX model, compared with Ti, the binding force between the new bone tissue connected with TiCu screw and the old bone tissue was tighter, hence more bone mass attached to the surface of TiCu screw when pulling out the screws, which resulted in a larger hole left. To sun up, the pull-out force of the screws during the removal process is determined by the joint strength of the screw–bone interface and the tearing force between the new bone tissue attached on the screw surface and the old bone tissue.

### Histological analysis

#### H&E and Masson staining

**Figure 5.**
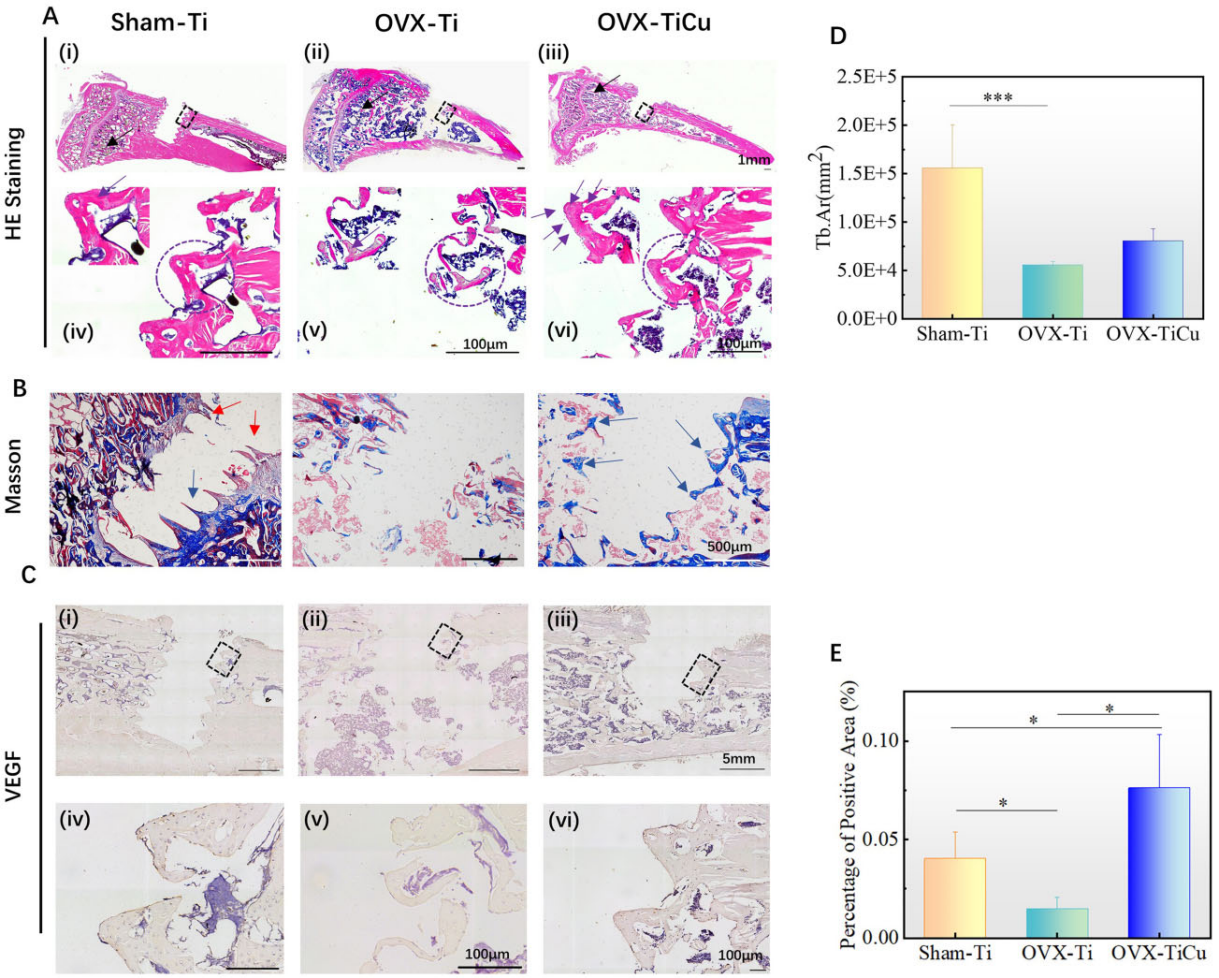
Analyses of soft tissue slices and immunohistochemical staining after 4 weeks of screw implantation: (**A** and **B**) soft tissue slices of H&E staining and Masson staining; (**C**) immunohistochemical staining of VEGF; (**D**) quantitative Tb. Ar around the screws; and (**E**) ratio of VEGF positive area to total bone area around the screws. Data were expressed as mean±SD. *n* = 7 in panels (D) and (E). ^*^P <* *0.05, ^***^P <* *0.001

The results of H&E staining were shown in Fig. 5A. The cells and extracellular matrix were purple blue, and the bone tissue was pink. Figure 5A(i–iii) shows the macro morphology of tibia, and Fig. 5A(iv–vi) was the enlarged views of inter-teeth area of the screw. Enlarging the contents in purple circles, as shown by the small pictures in the upper left corner of Fig. 5A(iv–vi), there were bone lacunae and bone cells in the bone trabecula (purple arrow). In OVX-Ti group, the bone mass and the number of osteocytes decreased significantly, the bone structure was incomplete, and there was only a small amount of bone tissues attaching to the diaphysis at implantation site of the screw. The bonding degree at the screw–bone interface was poor, which made the bone matrix in bone marrow cavity easily to be brought into the implant pits during the pull-out process of screw. The staining on screw edge was fuzzy and the distribution of bone cells was sparse, which reflected a lower degree of new bone formation and proved an obvious osteoporotic characteristic. In sharp contrast to OVX-Ti, OVX-TiCu group had more attached bone tissue between screw and diaphysis, increasing abundance of the vascular in metaphysis and medullary cavity (black arrow, Fig. 5A(iii)), and a large number of bone cells gathered at the bone-screw contact surface (purple arrow, Fig. 5A(vi)), indicating that TiCu screw significantly promoted the repair of osteoporotic bone structure after 4 weeks of implantation. The bone tissue of Sham-Ti group grew tightly with more bone mass, and the distributions of vascular tissue and bone matrix in bone marrow cavity were continuous. The bone trabeculae around the screw were well connected with the diaphysis, the vascular tissue evenly distributed at the metaphysis (black arrow, Fig. 5A(i)), and the bone cells around the screw evenly dispersed in the bone trabecular matrix. Quantitative analysis (Fig. 5D) showed that the Tb. Ar around the screw in OVX-TiCu group (0.08 ± 0.01 m^2^) was higher than that in OVX-Ti group (0.06 ± 0.004 m^2^), indicating that TiCu screw could promote new bone formation and its remodeling toward regular trabeculae to a certain extent after 4 weeks of screw implantation.

Masson staining results were shown in Fig. 5B, in which the collagen fibers and cartilage showed blue, the muscle fibers showed red and the nucleus showed blue black. The staining results revealed that there was almost no collagen around the screws in OVX-Ti group. Inversely, in OVX-TiCu group, there was continuous blue collagen distributed along the teeth edge of the screw (blue arrow) that grew into the cancellous bone. In Sham-Ti group, there was less collagen, and the inflammatory cells (red arrow) existed on the edge of screws.

Overall, after implantation for 4 weeks, TiCu alloy could not only significantly promote angiogenesis and repair of vascular tissues around the implantation site, but also promote osteogenesis, its remodeling toward trabecular structure around the screws.

#### Immunohistochemistry

VEGF, an important active growth factor in the process of angiogenesis, can directly regulate the formation of endothelial blood vessels. After implantation for 4 weeks, the expression of VEGF around the screw was analyzed by immunohistochemical staining. The positive reaction area showed brown yellow. The larger the brown yellow staining area is, the higher the expression of VEGF is, representing stronger proliferation and migration ability of vascular endothelial cells and higher vascular growth activity. As shown in Fig. 5C, all the groups showed positive expression of VEGF. Further, the quantitative analysis (Fig. 5E) showed that on both sides of the screw, the positive expression of VEGF in OVX-Ti group was the lowest (0.014% ± 0.005%), and that in OVX-TiCu group (0.080% ± 0.03%) was significantly higher than that in OVX-Ti group (*P *<* *0.05) and even higher than that in Sham-Ti group (0.040% ± 0.01%, *P *<* *0.05). This illustrates that after 4 weeks implantation, by up-regulating the expression of VEGF gene, TiCu screw could significantly promote the vascular growth and then accelerate the regeneration of vascular tissue around the implant.

#### Fluorescent labeling

**Figure 6.**
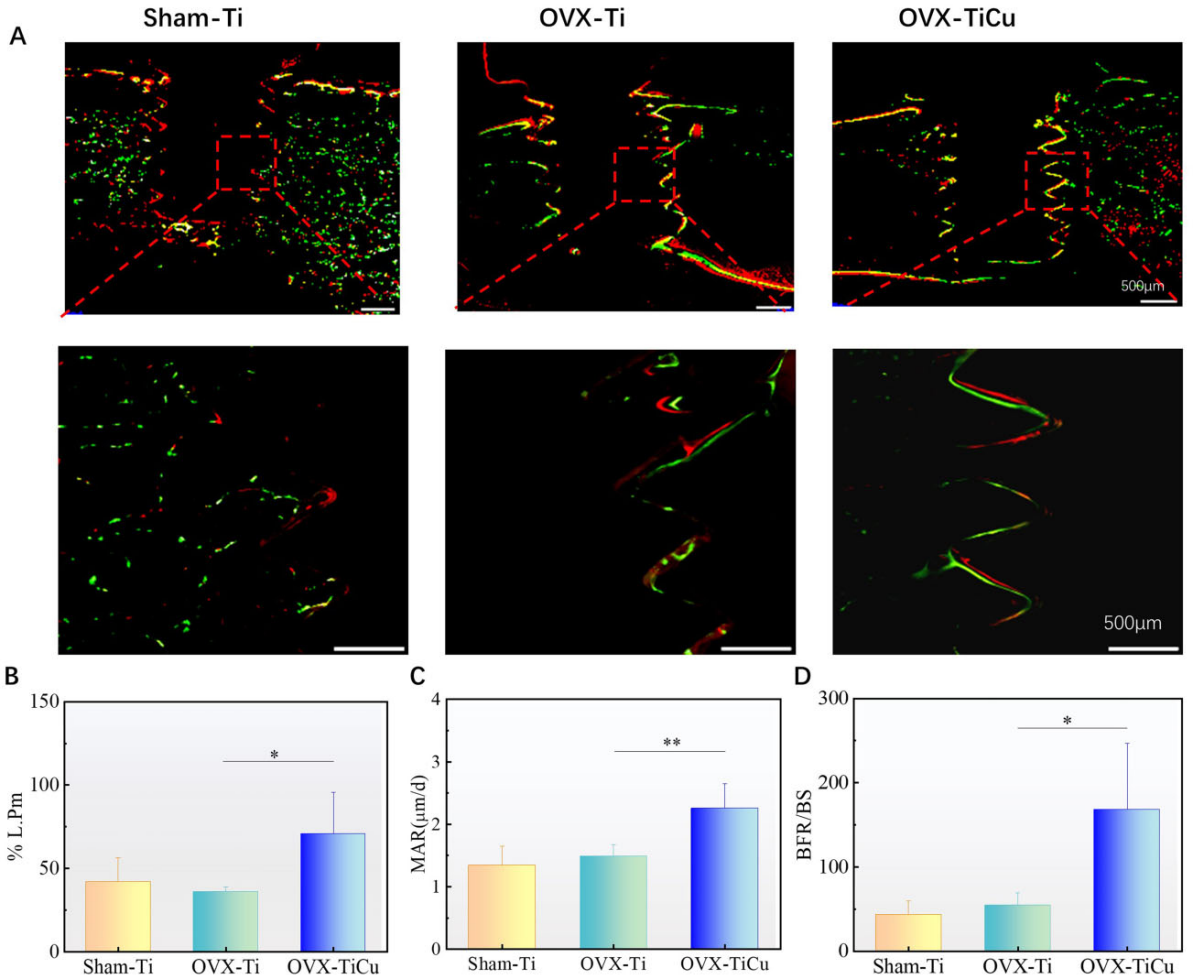
Analyses of hard tissue after screw implantation over 8 weeks: (**A**) hard tissue slices of fluorescence staining; and (**B** and **D**) quantitative analyses of OB activity. Data were expressed as mean±SD. *n* = 8 in panels (B) to (D). ^*^P <* *0.05, ^**^P <* *0.01

Xylenol orange and calcein can chelate with amorphous calcium phosphate (ACP) in bone, and the products are deposited at the front of bone mineralization, which can form red and green deposition zones, marking new bone formation. The more continuous and wider the fluorescence bands are, the higher the activity of new bone formation is, and the better the osseointegration is. After implantation for 8 weeks, as shown in Fig. 6A, there was double fluorescence development along the screw-bone boundary. Compared with OVX-Ti group, the double fluorescent markers in OVX-TiCu group were more complete, and the fluorescent bands were evenly distributed along the screw side, indicating that the new bones extended along the screw surface and there was an increased area of new bone-screw connection. However, the double fluorescent markers in Sham-Ti group were more dispersed, and the activity of new bone formation was significantly lower than that in OVX group.

In addition, the fluorescence perimeter percentage (%L.Pm), MAR and BFR/BS were also quantified. These data reflected the number of OBs around the screw, OB activity and bone formation on the bone surface, respectively. The higher the values of these data were, the higher the activity of bone formation was. The quantitative analysis (Fig. 6B–D) showed that the %L.Pm, MAR and BFR/BS of OVX-TiCu group were 70.67 ± 25.03, 2.25 ± 0.39 μm/d and 168.17 ± 78.35, respectively, much higher than those of OVX-Ti group (36 ± 2.94, 1.48 ± 0.19 μm/d and 54.25 ± 15.11) and Sham-Ti group (42 ± 14.04, 1.34 ± 0.31 μm/d and 43.2 ± 16.68). This indicated that in the OP model, after 8 weeks implantation, TiCu screw could largely improve the activity and promote the proliferation of OBs, so as to markedly enhance the bone formation activity and accelerate the growth and repair of bone tissue around the implant.

## Discussion

To solve the problems of implant loosening and longer healing time in the treatment of OPF, it is commonly expected to develop highly osteointegrated internal fixation implants. In this study, the experimental results of biomechanical properties, vascular regeneration and bone tissue remodeling around the implant confirmed that TiCu implant could significantly accelerate the process of osteointegration in the OVX modeled rats, with the assessments focused on the effects of TiCu screws implanted for different times on the surrounding vascular network, collagen fiber deposition and new bone formation in the OVX rats. The relevant underlying mechanisms of promotion effect of TiCu screw on angiogenesis, osteogenesis and remodeling of the new bone around the implant were also explored.

Before screw implantation (baseline), the rats after OVX operation have been confirmed to be developed to OP, and the vascular number of OVX groups decreased significantly due to the lack of estrogen, which down-regulated the expression of related vascular factors [32]. In the early stage of screw implantation (4 weeks), 3D imaging of vascular system (Fig. 2A) showed that the number of capillaries around the screws in OVX-Ti group was still sparse. There were few connected vessels, and the distribution of blood vessels was relatively discrete. In sharp contrast, in OVX-TiCu group, the distribution density of vascular network around the screw was significantly higher, implying that there were more new capillary networks around the screw, and the blood vessels extended to the implantation site and interacted with the screw. Thus, TiCu screws could significantly promote the reconstruction of vascular network, laying a beneficial foundation for bone formation.

In order to explore the mechanism of vascular network reconstruction with presence of TiCu screw in the osteoporotic environment, the expression of VEGF in the early stage of screw implantation (4 weeks) was analyzed by immunohistochemistry. In numerous growth factors, VEGF is considered to be the most common, effective and long-term signal to stimulate angiogenesis. It can induce the endothelial cell migration, proliferation, aggregation into tubes and enhance the vascular permeability by promoting the expression of vascular endothelial growth factor receptor [14, 15, 39]. Previous *in vitro* and *in vivo* studies on healthy animal models showed that Cu^2+^ could up-regulate the transcriptional activation of VEGF gene in vascular endothelial cells, promote endothelial cell migration and proliferation, increase vascular permeability, and thus promote the process of angiogenesis [40–42]. Early in 2002, Sen *et al.* [43] demonstrated that CuSO_4_ could induce VEGF expression in human keratinocytes at physiological concentrations by using Cu-based therapy. Gerard *et al.* [44] combined Cu^2+^ with VEGF and found that this combination significantly increased the complexity of HUVECs angiogenic network, and promoted wound healing and vascularization of tissues around implants in mouse model. Our previous study confirmed that TiCu alloy could obviously stimulate the expression of hypoxia-inducible factor (HIF-1α) and VEGF in human bone marrow mesenchymal stem cells *in vitro* [25]. The present results were consistent with the above studies. The *in vivo* immunohistochemical staining results in this study showed that the positive expression of VEGF on both sides of the screw in OVX-TiCu group was also significantly higher than that in OVX-Ti group. Compared with healthy rats, the vascular abundance of OVX rats decreased significantly because of the OP state of rat bones, meanwhile, the trauma of bone tissue caused by screw implantation made the hypoxia of rat tibial shaft more significant. It was reported in our previous study that the Cu atoms on the TiCu alloy surface could oxidize preferentially, segregate at the metal/passivation film interface, pass through the passive film through tunneling effect as a matter of priority, and dissolve into the solution at the passive film/solution interface as Cu ions [27, 28]. Therefore, in the state of OP, the Cu ions released at the initial stage of TiCu screw implantation would more easily stimulate the expression of VEGF, release the HIF-1α, promote the above hypoxia response, and further accelerate the proliferation and differentiation of endothelial cells. What’s more, due to the release of Cu ions at the passive film/solution interface, the concentration of Cu ions around the screw would be higher than that in diaphysis. This would induce vascular proliferation toward the screw implantation site and even interaction with the screw. In addition, it is worth noticing that the overexpression of VEGF would cause the excessive germination of blood vessels, resulting in the expansion of blood vessel area and high-density distribution of blood vessels, which would lead to changes in the mineralized deposition template and make the related ossification changes [10]. As for the average inner thickness distribution of blood vessels in the ROI, the quantitative analysis results (Fig. 2E) showed that there was no statistic difference between OVX-TiCu group and Sham-Ti group, indicating that the promotion of VEGF expression by TiCu alloy was appropriate.

Numerous studies have confirmed that in the process of bone repair, the vascular system in bone tissue can not only serve as a key transport medium to transport OBs, bone induced growth factor and immune cells [40, 45–47], but also attract Col I adhesion, so that it can be used as an early template for mineral deposition to regulate the bone tissue morphology, and fully mediate and direct anaphase osteogenesis [10]. In this study, the Masson staining results (Fig. 5B) showed that in OVX-TiCu group, with the support of developed vascular network, the continuous collagen fibers distributed along the teeth interface of screw and extended to the bone trabeculae in medullary cavity. However, in OVX-Ti group, due to the sparse distribution of blood vessels, the collagen expression between screw threads was low, and there was almost no new collagen at the implantation site. Therefore, in the osteoporotic animal model, TiCu alloy could promote the repair and regeneration of vascular structure at the early stage of implantation, and further promote the secretion and transformation of collagen fibers at the bone-screw interface, which would provide an effective basis for osteogenesis under long-term implantation.

In the process of bone reconstruction, angiogenesis and bone formation are the main factors mediating osteointegration in the osteoporotic bone environment. This study has confirmed that in the early stage of implantation, TiCu alloy in the osteoporotic rats could promote the formation of high abundance vascular network interacting with the implants, and thus promote the formation of continuous collagen at the contact interface between the screw and bone tissue. Based on the above results, a long-term osteogenic mechanism of TiCu screw in the osteoporotic bone environment, with high abundance vascular network formed in the early stage of implantation, was further explored.

After implantation for 8 weeks, results of Micro-CT analysis, biomechanical test and fluorescence staining of new bone formation showed that compared with pure Ti screw, TiCu screw significantly improved the main osteogenic indices (*P *<* *0.05), including osteogenic activity (%L.Pm, MAR and BFR/BS), BMD and osteointegration strength of the screw. Besides those, TiCu screw could also significantly increase the volume of new bone with high binding strength to the screw surface, i.e. the volume of cavity left after pull-out of the screw. It is well known that BMD is an important clinical index to evaluate the degree of OP. In this study, BMD was found to be obviously enhanced at the implant site in OVX-TiCu group, indicating that TiCu screw could effectively slow down the degradation of bone structure and reduce the severity of OP in the OVX rats. All these outcomes supported that TiCu screw possessed a high osteointegration performance, and thus improved the long-term implantation stability by increasing osteogenic activity, forming dense new bones at the screw-bone interface and enhancing BMD at the implantation site.

Andre *et al.* found that there was a coupling effect between angiogenesis and osteogenesis, where VEGF up-regulated the expression of BMP-2 in endothelial cells, induce ALP activity of primary OBs and promote osteogenic differentiation and matrix mineralization. Along with the process of bone formation, OBs can continuously express VEGF mRNA and promote VEGF secretion under the action of BMP-2, so as to promote the endothelial cell proliferation and angiogenesis [48]. Liang *et al.* [49–53] found that Cu^2+^ could up-regulate the Runx family transcription factor 2 signal pathway and jointly up-regulate the expression of osteogenic related genes, such as ALP, OPN, OCN and BMP-2, so as to coordinate the signal cascade in time and space and promote proliferation, differentiation and mineralization of BMSCs into OBs. In this study, TiCu screw group formed an advanced vascular network and promoted early osteogenesis by up-regulating the expression of VEGF around the screws in the early stage of implantation. The dense vascular network would transport osteogenic related proteins and relevant essential ions to the surrounding bone tissues, which would promote the proliferation, differentiation and mineralization of OBs, improve the bone self-reconstruction system and promote vascular development.

During the long-term implantation process, the angiogenesis process and osteogenesis process would complement each other in the osteoporotic environment, and could ultimately coordinate and improve the osteogenic ability of osteoporotic rats from many aspects. In addition, interestingly, as shown in Fig. 6A, there was a continuous double fluorescence staining along the thread edge of implanted screws in OVX-TiCu group and the fluorescent staining site was consistent with the distribution of collagen fibers stained by Masson at 4 weeks after screw implantation. This is because in the long-term bone remodeling process, collagen fibers can be used as a template for mineral deposition, which makes Ca^2+^ and PO4^3−^ ions nucleate in the gap area of fiber structure and forms ACP in the initial stage of mineralization process. ACP deposited at the interface between screw and bone could increase the bonding strength between TiCu screw and bone tissue [54, 55].

**Figure 7.**
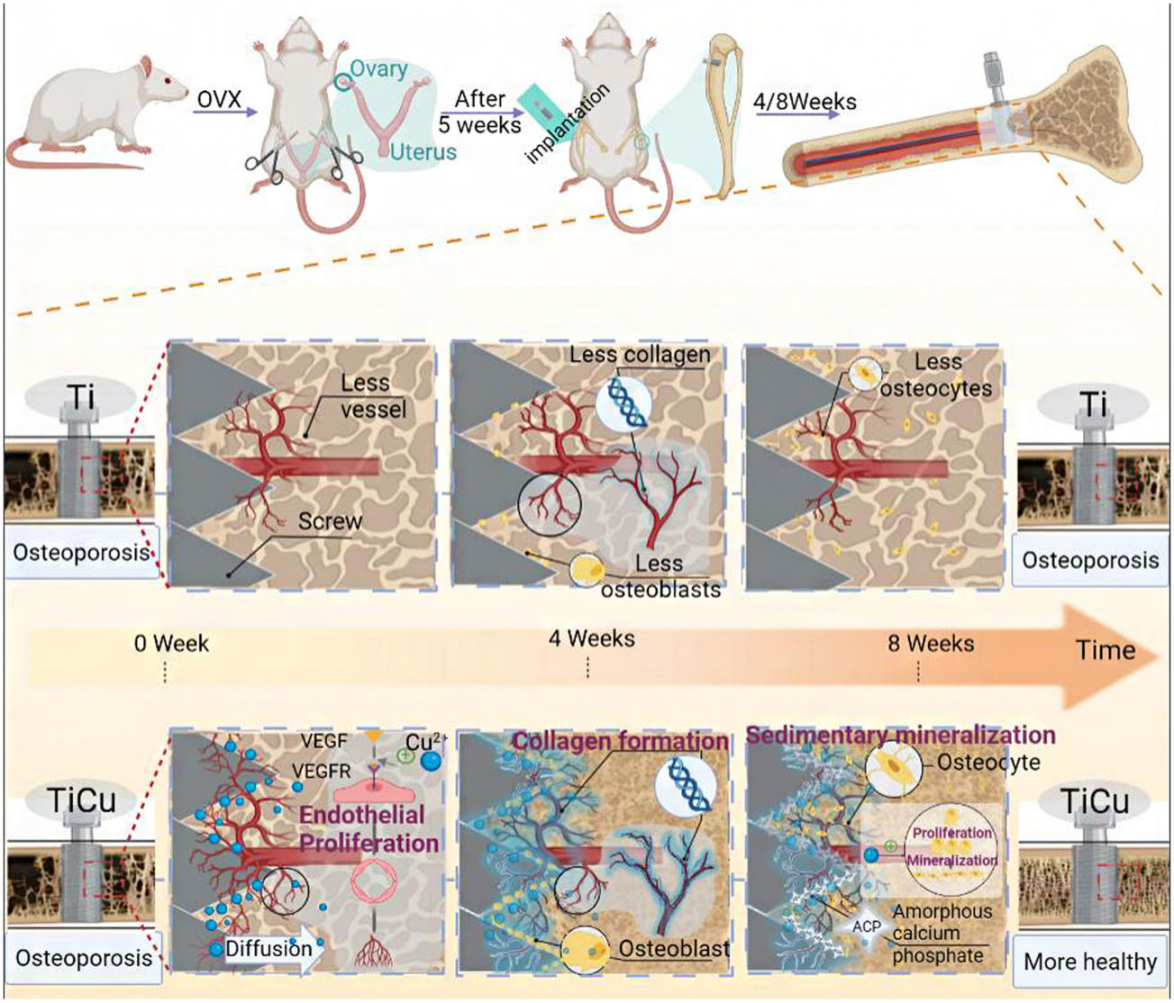
Illustration on different effects of TiCu and Ti screws implanted in tibia of osteoporotic rats

Through the above results and analyses, as shown in Fig. 7, it could be deduced that in the osteoporotic environment, TiCu alloy could up-regulate VEGF gene expression by releasing Cu ions in the early stage of implantation (4 weeks), which could promote formation of blood vessels and their network around the screw in favor of new bone formation. This not only provided sufficient nutrients for cells around the screw, but also induced the proliferation and differentiation of OBs around TiCu screw with extension of implant time (8 weeks). Accordingly, it promoted the mineralization and deposition of collagen on the surface of blood vessels, regulated the spatial distribution of new bone tissue and accelerated the process of bone formation. Under this multi-level coordination, TiCu screw could improve the compactness of new bone tissue around the implant, slow down the degradation of bone structure, enhance the binding between the screw and bone and ultimately grant a long-term stability of screw implantation.

## Conclusion

Compared with pure Ti screws, the TiCu screws presented much better osteointegration ability in the OVX modeled rats. TiCu screws could promote the reconstruction of surrounding vascular network at the early stage of implantation in the proximal tibial shaft of rats, adjust the spatial distribution of collagen and new bone tissue, and thus achieve a long-term osteointegration with promoting formation of new bone around the screw shaft. This study proved that TiCu alloy could effectively accelerate the formation of bone tissue, enhance BMD and reduce the risk of re-fracture in the osteoporotic bone environment, which has great potential to be used in the treatment of OPFs.
